# Interaction of OsRopGEF3 Protein With OsRac3 to Regulate Root Hair Elongation and Reactive Oxygen Species Formation in Rice (*Oryza sativa*)

**DOI:** 10.3389/fpls.2021.661352

**Published:** 2021-05-25

**Authors:** Eui-Jung Kim, Woo-Jong Hong, Win Tun, Gynheung An, Sun-Tae Kim, Yu-Jin Kim, Ki-Hong Jung

**Affiliations:** ^1^Graduate School of Biotechnology and Crop Biotech Institute, Kyung Hee University, Yongin, South Korea; ^2^Department of Plant Bioscience, Pusan National University, Miryang, South Korea; ^3^Department of Life Science and Environmental Biochemistry, and Life and Industry Convergence Research Institute, Pusan National University, Miryang, South Korea

**Keywords:** *Oryza sativa*, OsRopGEF3, OsRac3, OsRBOH5, reactive oxygen species, root hair

## Abstract

Root hairs are tip-growing cells that emerge from the root epidermis and play a role in water and nutrient uptake. One of the key signaling steps for polar cell elongation is the formation of Rho-GTP by accelerating the intrinsic exchange activity of the Rho-of-plant (ROP) or the Rac GTPase protein; this step is activated through the interaction with the plant Rho guanine nucleotide exchange factor (RopGEFs). The molecular players involved in root hair growth in rice are largely unknown. Here, we performed the functional analysis of *OsRopGEF3*, which is highly expressed in the root hair tissues among the *OsRopGEF* family genes in rice. To reveal the role of OsRopGEF3, we analyzed the phenotype of loss-of-function mutants of *OsRopGEF3*, which were generated using the CRISPR-Cas9 system. The mutants had reduced root hair length and increased root hair width. In addition, we confirmed that reactive oxygen species (ROS) were highly reduced in the root hairs of the *osropgef3* mutant. The pairwise yeast two-hybrid experiments between OsRopGEF3 and OsROP/Rac proteins in rice revealed that the OsRopGEF3 protein interacts with OsRac3. This interaction and colocalization at the same subcellular organelles were again verified in tobacco leaf cells and rice root protoplasts via bimolecular functional complementation (BiFC) assay. Furthermore, among the three respiratory burst oxidase homolog (OsRBOH) genes that are highly expressed in rice root hair cells, we found that OsRBOH5 can interact with OsRac3. Our results demonstrate an interaction network model wherein OsRopGEF3 converts the GDP of OsRac3 into GTP, and OsRac3-GTP then interacts with the N-terminal of OsRBOH5 to produce ROS, thereby suggesting OsRopGEF3 as a key regulating factor in rice root hair growth.

## Introduction

Root hairs are specialized cells formed by the expansion of epidermis cells located on the root’s outermost layer. They play an important role in supporting plant growth-like hormone response or absorbing water and nutrients (Gilroy and Jones, 2000; Vissenberg et al., 2020). The pattern of root hair cells (termed trichoblasts) and non-hair cells (atrichoblasts) is determined by their fate in the meristematic zone; the length of the root hair increases in the elongation zone of the root, while its growth stops in the maturation zone (Dolan et al., 1993; Duckett et al., 1994; Bibikova and Gilroy, 2002). Root hairs develop in a polarized manner from trichoblasts. Root hair growth involves cell wall and cytoskeleton reorganization, which is regulated by signaling molecules such as reactive oxygen species (ROS) and calcium gradients (Lin et al., 2015; Mangano et al., 2017).

Molecular genetic studies in *Arabidopsis thaliana* have identified key genes regulating root hair development. These include several transcription factors affecting the growth of root hair length, such as ROOT HAIR DEFECTIVE SIX-LIKE (RSL) transcription factor and R2R3 class MYB transcription factor (Slabaugh et al., 2011; Datta et al., 2015). Auxin response factor 5 (ARF5) regulates the expression of RSL4, and RSL2/RSL4 binds to the root hair-specific *cis*-element (RHEs) present in the promoters of RBOHC and RBOHH to produce ROS (Mangano et al., 2017). RBOHC and RBOHH loss-of-function mutants show reduced root hair length and ROS at the apical tip. ROS at the tip of the root hairs regulates the activity of calcium ion channels (Foreman et al., 2003; Carol et al., 2005; Jones et al., 2007), thereby regulating the activity of enzymes involved in cell wall expansion (Bosch and Hepler, 2005; Palin and Geitmann, 2012). Similarly, RSL and RBOH have been found in rice (*Oryza sativa*). OsRSL class II subfamily binds with rice root hairless 1 (OsRHL1) to regulate root hair growth (Moon et al., 2019b). Furthermore, auxin-responsive OsRBOH3 gene regulates root hair development (Wang et al., 2018).

In coordination with ROS, calcium, and cytoskeleton, a small GTP-binding protein, namely, ROP Rho-related GTPases from plants (ROP/Rac), acts as a molecular switch that cycles between an active (GTP-bound) and an inactive (GDP-bound) conformation (Cherfils and Chardin, 1999; Yang, 2002; Berken et al., 2005). Active ROP/Rac binds to the N-terminus of RBOH, after which the exposed EF-hand motif of RBOH (Wong et al., 2007) can bind with calcium ions. Disruption of ROP affects apical actin dynamics, calcium signaling, and ROS production, and constitutive activations also alter tip growth, resulting in wavy or swollen root hair tips (Molendijk et al., 2001; Bloch et al., 2005).

Recently, the molecular player targeting ROPs to the polarized tip membrane was identified as ROP-guanine nucleotide exchange factor (RopGEF), which converts inactive ROP to active ROP. Among the 14 RopGEF genes in *Arabidopsis* (Gu et al., 2006), AtRopGEF3 regulates the initiation and bulging of root hair through physical interaction with AtROP2, while RopGEF4 regulates subsequent growth of root hair tips (Denninger et al., 2019), indicating the subfunctionalization of different RopGEF members. The molecular function of RopGEF and ROP/Rac in root hair growth of other plants is less known. There are 11 *OsRopGEFs* and 7 *OsRac* genes in rice (Miki et al., 2005; Kim et al., 2020). The functions of control grain size, RNA silencing, apoptosis regulation, and gene expression in OsRacs have been studied (Kawasaki et al., 1999; Miki and Shimamoto, 2004; Zhang et al., 2019). Phylogenetic analysis and expression profiles suggest different features of ROP between *Arabidopsis* and rice (Kim et al., 2020). However, it has not been elucidated how OsRopGEF and OsRac regulate root hair development in rice, a model crop plant.

In this study, we aim to elucidate the function of OsRopGEF3 in rice root hair growth. First, we identified how OsRopGEF3 affects root hair development in rice. Using the CRISPR-Cas9 gene editing system, we found that the loss-of-function mutant of OsRopGEF3 showed an abnormal root hair phenotype, with ROS reduction. Furthermore, the OsRopGEF3 protein localizes at the plasma membrane (PM)-associated cytoplasm within rice root hair cells. Through yeast two-hybrid (Y2H) and bimolecular fluorescence complementation (BiFC) analyses, we found that OsRopGEF3 interacts with OsRac3. Finally, we identified that the N-terminal of OsRBOH5 interacts with OsRac3. We also describe and discuss the mechanism of rice root hair development associated with OsRopGEF3, OsRac3, and OsRBOH5.

## Materials and Methods

### Multiple Sequence Alignment, Meta-Expression Analysis, and Domain Analysis

To perform a phylogenetic analysis of the OsRac family, protein sequences were collected from the rice genome annotation project^1^ (Moon et al., 2020). Multiple amino acid sequences were aligned using ClustalW, and phylogenetic analysis was performed using MEGA-X under neighbor-joining methods. Using a publicly available rice Affymetrix microarray data in the National Center for Biotechnology Information Gene Expression Omnibus (NCBI GEO datasets), we normalized the signal intensity with the R language and then transformed them into log2 values. The normalized data, with averaged Affymetrix anatomical meta-expression data, were used for heatmap construction. The TMHMM^2^ and Pfam^3^ databases were used for domain analysis.

### Plant Growth, Nucleic Acid Extraction, and RT-qPCR

To grow rice (*O. sativa japonica* cv. Dongjin), the seeds were sterilized with 50% of sodium hypochlorite for 30 min, washed with distilled water for three times, and then germinated on Murashige and Skoog (MS) media under controlled conditions in 7 days (28/25°C day/night, 8-h photoperiod, and 78% relative humidity). The seedlings were grown in the growth chamber or greenhouse for 1 month and then transferred to a paddy field of Kyung Hee University. The tobacco plants (*Nicotiana benthamiana*) were grown in chambers at controlled conditions (25°C day/night, 16-h photoperiod, and 50% relative humidity) in 3–4 weeks.

Various rice tissues were immediately frozen in liquid nitrogen and ground with Tissue-Lyser II (Qiagen, Hilden, Germany) or mortar and pestle (CoorsTek 60310). The leaf tissues were sampled from 1-month-old plants for mutant analysis, and six other tissues were sampled for real-time quantitative PCR (RT-qPCR) (shoots and roots from 1-week-old plants, leaves and young panicles from 1-month-old plants, developing seeds 5–10 days after pollination, and root hairs from seedling roots 3 days after germination). DNA was extracted using the cetyltrimethylammonium bromide (CTAB)-chloroform method, and the sequence genotype was analyzed in Macrogen Corp.^4^ using BigDye Terminator v3.1 cycle sequencing kit (Applied Biosystems). Total RNA was extracted with a TRIzol buffer and purified with an RNeasy plant mini kit (Qiagen); complementary DNA (cDNA) was synthesized using SuPrimeScript RT premix (GeNet Bio). To identify the tissue specific expression by RT-qPCR, we used the Roter-Gene Q instrument system (Qiagen, Hilden, Germany) and the internal control for rice ubiquitin 5 (*OsUbi5*, LOC_Os01g22490), as previously reported (Kim et al., 2019). RT-qPCR was performed with three independent biological replicates. Relative transcript levels and fold change were calculated by previously reported methods (Schmittgen and Livak, 2008). All RT-qPCR primers used in our experiments are listed in Supplementary Table 1.

### Vector Construction

To design the guide RNA for CRISPR-Cas9 vector cloning, we selected two target regions using the CRISPRdirect software (Naito et al., 2015). For CRISPR-Cas9 vector cloning, we synthesized oligo dimers with annealed primers and ligated the dimers with the pRGEB32 binary vector (Xie et al., 2015). To clone the *OsRopGEF3* promoter, the pOsRopGEF3:OsRopGEF3-GFP plasmid was generated; the 2000 base-pair upstream from the start codon and the open reading frame (ORF) of *OsRopGEF3* genes were amplified by PCR and cloned into the binary vector pGA3427, which includes the enhanced green fluorescent protein (eGFP) sequence. To clone the beta-glucuronidase (GUS) fusion vector (*pOsRopGEF3: GUS*), the 2000 upstream sequence from the stop codon was amplified and ligated into a BamH1-treated pGA3519 vector. Ligated vectors were transformed into *Escherichia coli*, *TOP10*. The confirmed plasmid was then transformed into *Agrobacterium tumefaciens*, *LBA4404*. The transgenic rice plants were generated via *Agrobacterium*-mediated cocultivation of the rice callus (Lee et al., 1999). All of the cloning primers we have used in our experiments are listed in Supplementary Table 1. For the protoplast experiments, the ORFs of *OsRopGEF3*, *OsRac3*, and *OsRBOH5* were each ligated into the Sma1-treated pGA3574 vector, which was tagged with eGFP, mCherry, and N- and C-terminal fragments of Venus.

### Morphological Analysis

To minimize the transformation side effects, at least three generations after the tissue culture of plants were used for all root hair measurements. All root hairs were measured from the primary roots of rice 3 days after germination (DAG). The root hair lengths and widths were quantified 2–8 mm from the root apex. The length was measured only when the starting point of the root hairs was visible from the root, and the width was measured at the center of the root hair. BX61 (Olympus, Tokyo, Japan) and SZX61 (Olympus, Tokyo, Japan) microscopes were used for plant photography, and the root hair lengths and widths were measured using Image J software (Schneider et al., 2012). Data were observed in 10 seedlings each for the control and mutant plants and presented as means and standard deviation.

### Histochemical Assay

For GUS staining, transgenic plants were mixed with GUS staining solution and vacuumed for 30 min. After that, samples were incubated for 24 h at 37°C in dark conditions (Moon et al., 2019a). Chlorophyll was gradually removed by 70% and absolute ethanol. For ROS detection, the primary roots from three DAG plants were collected and mixed with 10 μM 2′, 7′-dichlorodihydrofluorescein diacetate (CM-H2DCFDA) (Invitrogen) or 1 mg/ml 3,3′-diaminobenzidine (DAB) solution. Next, the samples were vacuumed for 15 min in dark conditions, followed by incubation for 30 min for CM-H2DCFDA staining and 3 h for DAB staining at room temperature in dark conditions. After staining, the primary roots were washed three times with 1% phosphate-buffered saline (PBS). Root hairs were observed under a BX61 microscope (Olympus) and a laser scanning confocal microscope LSM 510 (Carl Zeiss, Jena, Germany). GFP intensity was calculated using ZEN Blue software.

### Y2H Analysis

The full-length coding sequence (CDS) of OsRopGEF3 and the N-terminus region of OsRBOH5 were fused into a pGADT7 vector, and the full CDS of OsRac3 was fused with the pGBKT7 vector. Each construct was transformed into the AH109 strain and plated on SM media lacking leucine, tryptophan, histidine, and alanine and incubated for 3 days at 30°C as previously reported (Fields and Song, 1989). The primer sequences used in Y2H analysis are listed in Supplementary Table 1.

### Subcellular Localization Analysis

The ORFs of OsRopGEF3 and OsRac3 were amplified from the root hair cDNA and cloned into a pGREEN vector fused with GFP. For BiFC, Venus fluorescence protein, which emits a bright yellow signal, was used for complementation. OsRopGEF3 was fused with the N-terminus of Venus, and OsRac3 was fused with the C-terminus of Venus.

The constructs were transfected into the *A. tumefaciens* strain GV3101 and infiltrated in tobacco leaf as described previously (Sparkes et al., 2006). All constructs used for tobacco infiltration were expressed under the control of the Cauliflower mosaic virus (CaMV) 35S promoter. After 72 h of infiltration, GFP fluorescence was observed by a confocal laser scanning microscope (Zeiss LSM 510, Jena, Germany) with 500–530 nm for emission and 488 nm for excitation. YFP fluorescence was detected at 530–560 nm for emission and 500–530 nm for excitation. FM4-64 (Thermo Fisher Scientific) was used as a PM marker and observed with red fluorescence protein (RFP) channel in a 558-nm microscope. The empty GFP protein was used as a control (Supplementary Figure 2).

For protoplast transformation, OC cells isolated from rice root cells were cultured in R2S media for at least 1 month. The protoplasts were then extracted using cellulase and macerozyme r-10 (Yakult) as previously reported (Cho et al., 2016; Wong et al., 2018). The construct was transformed using PEG-mediated protoplast transfection. After transformation, the protoplasts were incubated for >16 h in dark conditions. All the primers used in our experiments are listed in Supplementary Table 1.

## Results

### Identification of Root Hair-Specific Genes Expressed in Rice

**FIGURE 1:**
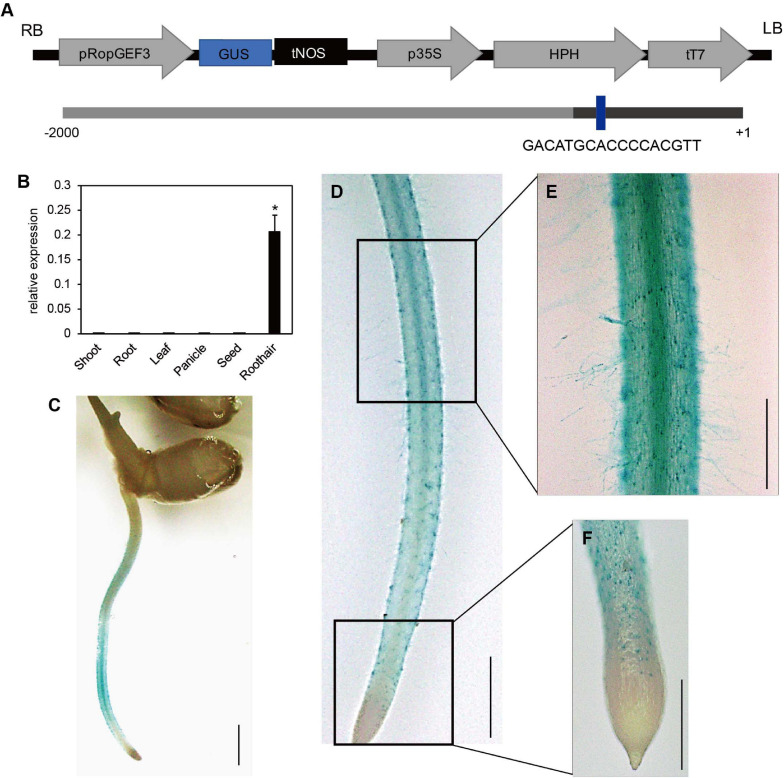
Expression pattern of *OsRopGEF3.* **(A)** The pGA3519 vector map used for histochemical GUS assay of OsRopGEF3 genes. The pGA3519 vector contains a sequence of hygromycin-resistance genes. LB, left border of the vector; RB, right border of the vector; pOsRopGEF3, promoter sequence of *OsRopGEF3*; GUS, beta-glucuronidase; tNOS, NOS terminator; p35S, 35S promoter; HPH, Hygromycin-B-phosphotransferase gene; tT7, T7 terminator. The following shows the promoter region of OsRopGEF3. The dark bar represents the 5′-untranslated region (UTR). The RHE cis-element region is present in the promoter of OsRopGEF3. + 1 means the first base of the start codon, and -2,000 means 2,000 base pairs upstream from it. **(B)** The real-time quantitative PCR (RT-qPCR) analysis of OsRopGEF3 from diverse rice tissues. Rice ubiquitin 5 gene (*OsUBI5*; LOC_Os01g22490) was used as an internal expression control. *X*-axis, the sample name used for analysis; *Y*-axis, the relative expression level of OsRopGEF3. The error bar indicates standard errors in three biological replicates. Significant differences are indicated by asterisks, *p < 0.01. The data were analyzed using one-way ANOVA with repeated measurements using Tukey’s pairwise comparison test. **(C–F)** Histochemical GUS assay of transgenic lines. The 2,000 base-pair upstream region from the start codon of OsRopGEF3 genes cloned and ligated to the pGA3519 vector. **(C)** The picture of whole plants, bars = 3 mm. **(D)** The entire root from the apical tip to the maturation zone, bars = 2 mm. **(E)** The root hairs and the elongation zone of root, and **(F)** the apical tip and meristematic zone of the root, bars = 500 μm.

The meta-analysis of public microarray data based on the National Center for Biotechnology Information Gene Expression Omnibus (NCBI GEO)^5^ datasets indicated that *OsRopGEF1*, *OsRopGEF3*, and *OsRopGEF11* of the *OsRopGEF* family genes are highly expressed in the root hair tissue (Kim et al., 2020). Among them, *OsRopGEF3* is also strongly expressed in reproductive tissues, such as anthers and pollen, and has high sequence similarity to pollen specific *OsRopGEF* genes. Based on the expression pattern and sequence similarity analyses, we assumed that OsRopGEF3 might have a more important function for root hair elongation than the others because pollen tubes and root hairs share tip-focused growth. RT-qPCR and histochemical assay using GUS proteins have verified the expression patterns of *OsRopGEF3* (Figure 1). *OsRopGEF3* was expressed more in root hair tissues than in other tissues (Figure 1B). In addition, RHE lies upstream of OsROPGEF3 (Figure 1A), indicating possible gene expression regulated by RSL in a root hair-specific manner. In rice seedlings at three DAG, the GUS signal of *OsRopGEF3* was strongly observed in the root hair tissues and elongation zone of the root, where the root hairs usually start to grow (Figures 1C,D). GUS signals are mainly observed in the trichoblast cells of the meristematic zone and the root hairs in the elongation zone (Figures 1E,F).

### OsRopGEF3 Protein Is Mainly Localized in the PM of Rice Root Hairs

**FIGURE 2:**
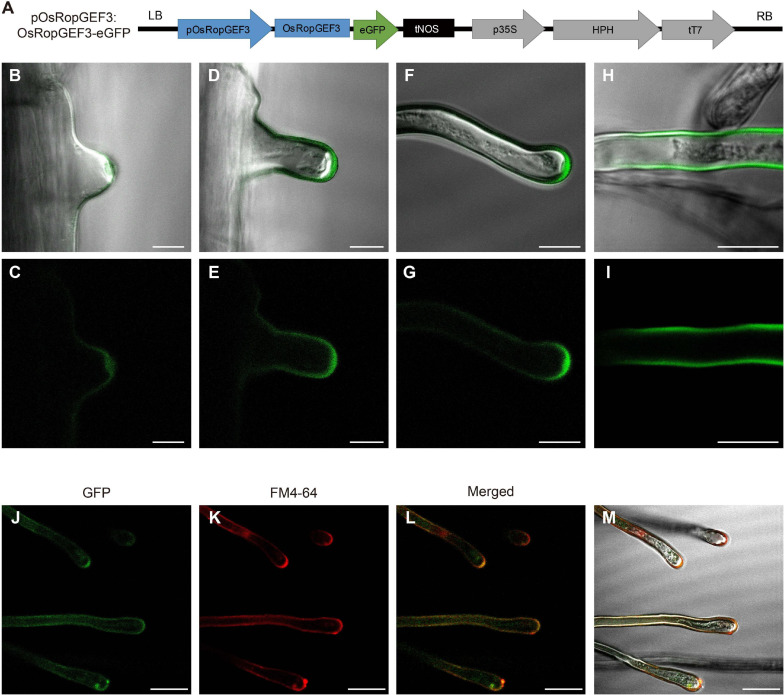
Subcellular localization of OsRopGEF3 in the growing root hairs in rice. **(A)** The pGA3427 vector map used for gain-of-function and fluorescent tagging of OsRopGEF3 genes. OsRopGEF3 native promoter, 2,000 base pairs upstream from the start codon, allowed it to be overexpressed only on the root hairs and tagged with an eGFP (pOsRopGEF3:OsRopGEF3-eGFP). The pGA3427 vector contains a sequence of hygromycin-resistance genes. LB, left border of the vector; RB, right border of the vector; pOsRopGEF3, promoter sequence of *OsRopGEF3*; eGFP, enhance green fluorescence protein; tNOS, NOS terminator; p35S, 35S promoter; HPH, hygromycin-B-phosphotransferase gene; tT7, T7 terminator. **(B–I)** The confocal images of rice root hair in transgenic lines (pOsRopGEF3:OsRopGEF3-eGFP). As root hairs gradually grow, the subcellular localization of OsRopGEF3 protein was detected by fluorescence. **(B,C)** The apical tip part of the initiation states, **(D–G)** the apical tip part of the elongation states, **(H,I)** fully grown root hairs, and **(J–M)** representative confocal images of the root hair in transgenic line stained with FM4-64 dye. FM4-64 was checked on the RFP channel. Bars = 10 μm **(B–I)** and 20 μm **(J–M)**.

To identify the subcellular localization of the OsRopGEF3 protein, we generate gain-of-function transgenic lines that express OsRopGEF3 protein fused with GFP under the native promoter (*pOsRopGEF3:OsRopGEF3-GFP*) in a wild-type (*O. sativa japonica* cv. Dongjin) background (Figure 2A). The OsRopGEF3 tagged with GFP protein localizes strongly at root hairs. When the development of root hair cells initiates, the GFP signal is mainly clustered and located in the cytoplasm of the tip region (Figures 2B,C). In the growing root hairs, the GFP signal was spread near the PM but was mainly located at the apical tip region (Figures 2D,E). The signal is getting stronger as the root hairs keep growing (Figures 2F,G). When the root hairs were fully grown, the fluorescent signals were distributed throughout the PM (Figures 2H,I). To verify this localization pattern, we stained the root hairs with FM4-64 dye, which has been used to track the PM. As a result, it was confirmed that the GFP signal appears PM-related in the root hairs through colocalization with FM4-64 signals (Figures 2J–M). It was colocalized in the PM, strongly observed in the root hair tip, and identified in the root hair body. However, when predicting protein domains through transmembrane helices hidden Markov models (TMHMM) and Pfam databases (hidden Markov models), no transmembrane domain was observed in OsRopGEF3 (Supplementary Figure 1). Of the 514 total amino acids in OsRopGEF3, S71 to K416 were aligned to the plant-specific ROP nucleotide exchanger (PRONE) domain.

### Abnormal Root Hair Phenotypes in the Loss-of-Function Mutant of OsRopGEF3

**FIGURE 3:**
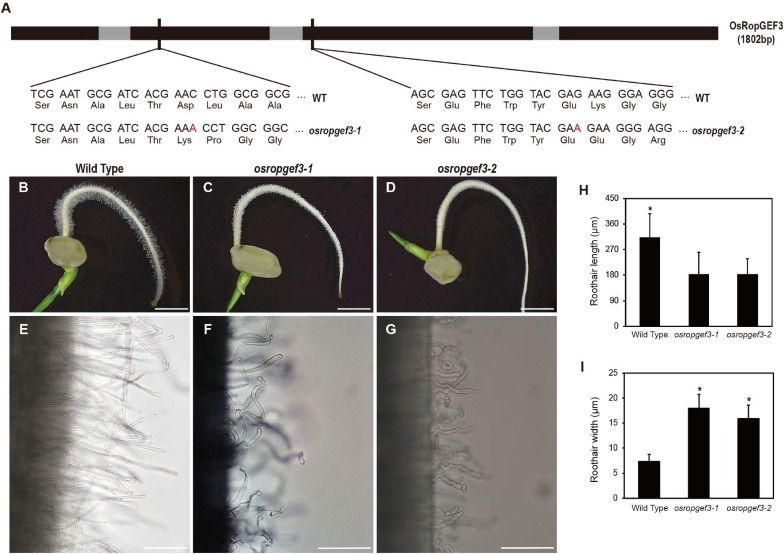
The functional analysis of OsRopGEF3 genes using loss-of-function on rice root hairs. **(A)** The black bar represents the exon, the gray bar represents the intron, and the thin black bar represents the gRNA target point for the mutant line. *OsRopGEF3* genomic DNA has 1802 base pairs. The first loss-of-function mutant (*osropgef3-1*) designated the front of the second exon as the gRNA target site, and the second mutant (*osropgef3-2*) designed the target in the third exon. In both mutant lines, one base (adenine) was inserted, and subsequently, both amino acid sequences were broken, and there was no stop codon produced. **(B–G)** Phenotypic analysis in 3 days after germination loss-of-function transgenic plants. Bars = 3 mm **(B–D)** and 100 μm **(E–G)**. **(H,I)** The root hair length and width comparison between the wild-type and *osropgef3* mutant. The length and width of the root hairs were quantified 2–8 mm from the root apex. The average was calculated by measuring at least 100 root hairs per plant. Error bars represent standard errors in six biological replicates. Significant differences are indicated by asterisks, *p < 0.05. The data were analyzed using Student’s *t*-test.

To know the functional roles of the gene, we generated the loss-of-function mutants of *OsRopGEF3* via targeted mutagenesis using the CRISPR-Cas9 system. We then identified two homozygous lines carrying mutations in different exon regions. The second and third exon of the *OsRopGEF3* were targeted for gene editing, and corresponding mutants are named, *osropgef3-1* and *osropgef3-2* (Figure 3A). The *osropgef3-1* line has one base (adenine) insertion in the second exon, and the *osropgef3-2* line has one base (adenine) insertion in the third exon. Both lines have abnormal protein sequences, which change the ORFs but do not create a stop codon. All loss-of-function mutant lines produce defective root hair development phenotypes. Differences in root hair development between mutant and wild types can be clearly distinguished from three DAG (Figures 3B–D). Phenotypic differences were the greatest in plants on 3–5 DAG. To compare the phenotypes in detail, we measured the length and width of root hairs between the wild-type and *osropgef3* mutants at three DAG. On average, wild-type root hairs were 290 μm long and 7 μm thick (Figures 3H,I). In contrast to wild-type root hairs, the length of the root hairs of *osropgef3-1* and *osropgef3-2* decreased by 180 μm, while the width increased up to 16–18 μm and showed a wavy phenotype (Figures 3E–I), clearly presenting defects in root hair elongation.

### *OsRopGEF3* Is Involved in ROS Generation

**FIGURE 4:**
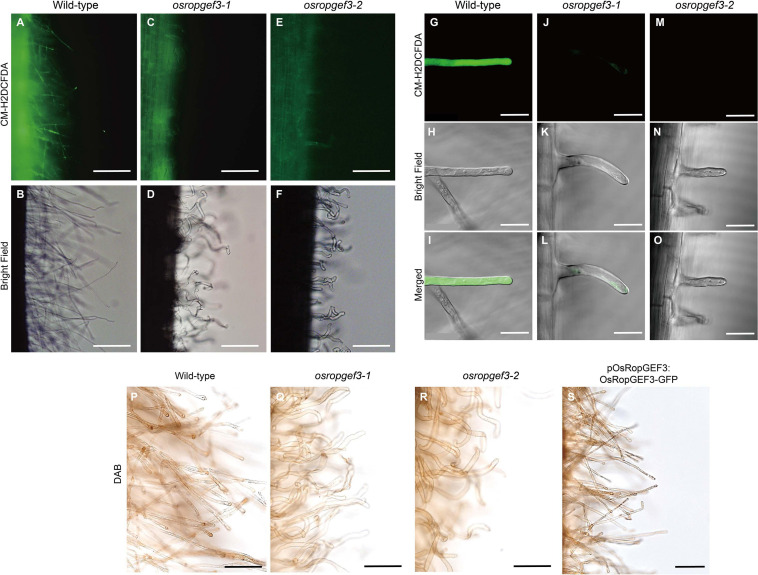
The difference in reactive oxygen species (ROS) in the root hairs of wild-type, *osropgef3* mutants, and gain-of-function line. Representative confocal images of the wild-type and OsRopGEF3 mutant root hairs. **(A–F)** Hydrogen peroxide staining using CM-H2DCFDA dye in three DAG rice roots. Bars = 100 μm. **(G–O)** Zoom photo of CM-H2DCFDA staining using a confocal microscope. Bars = 20 μm. **(P–S)** 3,3′-Diaminobenzidine (DAB) staining in wild-type, two loss-of-function homozygous mutants, and gain-of-function lines (pOsRopGEF3:OsRopGEF3-GFP) in the primary root of three DAG rice plants. Bars = 50 μm.

To check the intracellular levels of ROS generation in root hair cells, primary roots were stained with CM-H2DCFDA and DAB at three DAG (Figure 4). After CM-H2DCFDA staining, the wild-type plants were strongly stained throughout the roots (Figures 4A,B). However, the roots of *osropgef3-1* and *osropgef3-2* mutants were less stained, with negligible staining in the root hairs (Figures 4C–F). We then observed the root hairs using a confocal microscope to detect differences in ROS levels. Using the same fluorescence intensity, ROS was detected throughout the entire root hairs of the wild-type plants, but *osropgef3* mutants had weak signals in the apex or were not stained at all (Figures 4G–O). The GFP intensity of the root hair apex was approximately 3,500 for the wild-type plants and 0–500 for the mutants (Supplementary Figure 2). Next, to confirm the variation in ROS intensity in the OsRopGEF3 gain-of-function transgenic line (*pOsRopGEF3:OsRopGEF3-GFP*), we used DAB dye for staining. CM-H2DCFDA dye could not be used because this transgenic line was tagged with GFP. The wild-type root hairs were strongly stained at the apex region (Figure 4P), whereas *osropgef3* root hairs were weakly stained (Figures 4Q,R). The gain-of-function line showed staining similar to the wild-type plants (Figure 4S). These results demonstrate that *OsRopGEF3* plays a role in ROS production in root hairs.

### OsRopGEF3 and OsRac3 Interact in the PM

**FIGURE 5:**
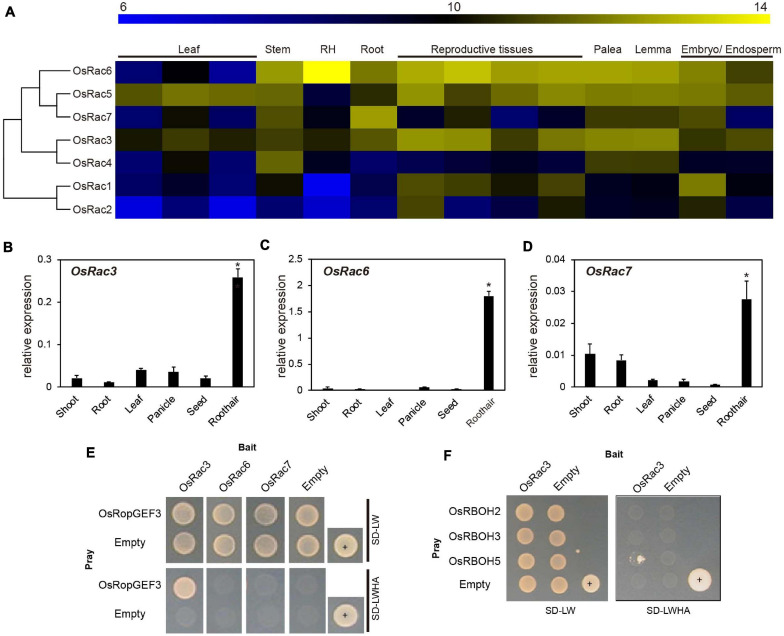
Expression pattern of OsRac and identification of the interacting proteins using yeast two-hybrid analysis. **(A)** Microarray meta-expression analysis and phylogenetic tree of seven *OsRac* genes. The phylogenetic tree was constructed by neighbor-joining methods. In the heatmap, the yellow color indicates a high expression, and the blue color indicates a low expression. Numeric values indicate an average of the normalized log2 intensity of microarray data. Leaf includes the leaf blade, leaf sheath, flag leaf, and reproductive tissues including the anther, carpel, ovary, and pistil. RH, root hair. **(B–D)** Expression verification of *OsRac* using real-time quantitative PCR (RT-qPCR). Rice ubiquitin 5 (LOC_Os01g22490) was used as an internal control. *X*-axis, the sample name used for analysis; *Y*-axis, the relative expression level of the *OsRac3*, *OsRac6*, and *OsRac7*. The error bar indicates standard errors in three biological replicates. Significant differences are indicated by asterisks, *p < 0.01. The data were analyzed using one-way ANOVA with repeated measurements using Tukey’s pairwise comparison test. **(E)** The interaction between OsRopGEF3 and three OsRac (OsRac3, OsRac6, and OsRac7) evaluated using the yeast two-hybrid system. **(F)** Identification of the interaction between OsRac3 and three OsRBOH that have highly specific expression in root hair tissues. SD-LW, synthetic defined media without Leu and Trp; SD-LWHA, synthetic defined media without Leu, Trp, His, Ala; +, positive control; empty vector of pGADT7 and pGBKT7 used as a negative control.

To find the functional interaction partner for OsRopGEF3 among the OsRac family members in the rice genome, we first analyzed the expression patterns of the entire *OsRac* genes. Heatmap expression data based on the public Affymetrix microarray data consisting of various rice tissues and organs were generated and integrated into a phylogenetic tree (Figure 5A). The meta-expression analysis suggests some candidate OsRac that have the potential to interact with OsRopGEF3 in root hairs. Of the seven *OsRac* genes, *OsRac6* has the highest expression in the root hair tissue, followed by *OsRac3*. *OsRac4* and *OsRac7* have weak expression in root hair. However, none of the *OsRac* genes showed strong expression only in the root hairs. Besides root hairs, *OsRac6* was also highly expressed in the stem, root, palea, lemma, embryo, endosperm, and all reproductive tissues. *OsRac3* was evenly expressed in all analyzed tissues and organs, and *OsRac4* was expressed in the stem, palea, and lemma. RT-qPCR results for six tissues, i.e., shoot, root, leaf, panicle, seed, and root hair, tended to be similar to meta-expression data patterns (Figures 5B–D). As expected, three genes, *OsRac3*, *OsRac6*, and *OsRac7*, showed a significant level of expression in root hairs. In particular, *OsRac6* showed the highest expression in root hair tissues. The expression of the remaining genes, i.e., *OsRac1*, *OsRac2*, *OsRac4*, and *OsRac5*, was negligible in the root hairs (Supplementary Figure 3).

**FIGURE 6:**
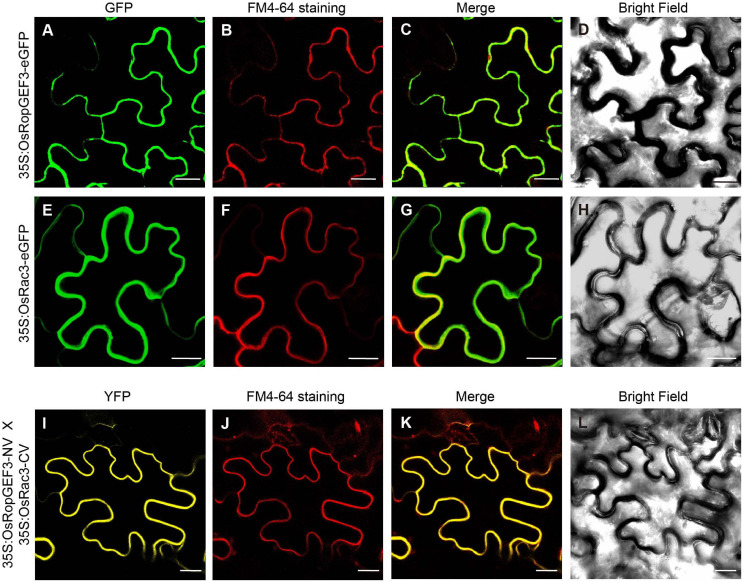
Subcellular localization and BiFC of OsRopGEF3 and OsRac3 in tobacco leaf cells. **(A–H)** Subcellular localization of OsRopGEF3 and OsRac3. These proteins were tagged with enhanced green fluorescent protein (eGFP) and transiently expressed individually in tobacco (*Nicotiana benthamiana*) leaf cells using a 35S promoter. The cells were stained with FM4-64 dye to confirm PM localization. FM4-64 is detected as red fluorescence protein (RFP) signals. **(I–L)** BiFC visualization of OsRopGEF3 and OsRac3. OsRopGEF3 was tagged with the N-terminus of the Venus protein, and OsRac3 was tagged with the C-terminus. Both constructs were coexpressed inside the cells and were stained with FM4-64 dye to confirm their localization. BiFC signals were detected as YFP signals. Bars = 20 μm.

**FIGURE 7:**
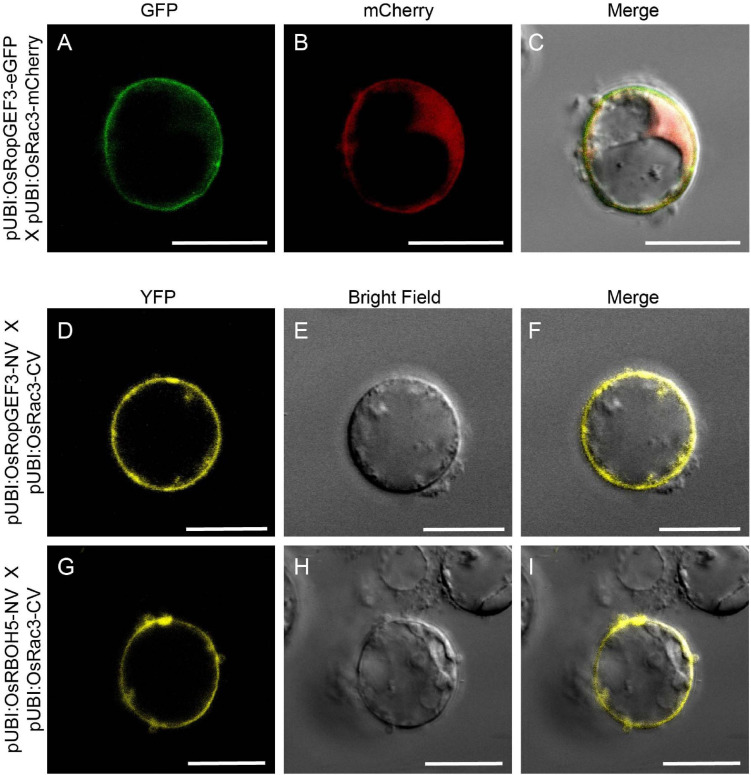
Colocalization and BiFC visualization in rice root protoplasts. **(A–C)** Subcellular localization of OsRopGEF3 and OsRac3. The complete ORF OsRopGEF3 was combined with eGFP, whereas that of OsRac3 was combined with mCherry. These constructs were coexpressed in root protoplasts. **(D–F)** Fill-length OsRopGEF3 and OsRac3 proteins were individually fused with the N- and C-terminals of Venus protein. **(G–I)** The N-terminal protein region of OsRBOH5 was fused with the N-terminal of Venus protein and cotransfected with OsRac3-CV. Each construct was expressed using a maize Ubi (ZmUbi) promoter. The protoplasts were observed under a laser scanning confocal microscope. Bars = 10 μm.

Using Y2H, we confirmed the interaction between OsRopGEF3 and these three OsRac proteins (Figure 5E). The full CDS of each protein was fused with the prey and bait domain. As a result, OsRac3 interacted with OsRopGEF3. However, although OsRac6 was most strongly expressed in root hairs, it did not show any interaction with OsRopGEF3. The interaction between OsRopGEF3 and OsRac3 has also been verified in tobacco leaf cells. The empty GFP vector was used as a positive control (Supplementary Figure 4). As shown in Figure 6, when expressed individually, OsRopGEF3 was located near the PM (Figures 6A–D), and OsRac3 was located in the PM and the cytoplasm (Figures 6E–H). FM4-64 dye was used as a PM marker. When 1 M NaCl treatment was used to cause plasmolysis, the GFP signals were merged well with FM4-64 (Supplementary Figure 5). Next, the interaction of these two proteins was verified within tobacco cells using BiFC. Similar to the Y2H results, we confirmed that OsRopGEF3 and OsRac3 bind near the PM (Figures 6I–L). Interestingly, OsRac3 was spread out in the cytoplasm when expressed alone, but with its binding partner OsRopGEF3, it was also localized near the PM. Intracellular colocalization of the proteins was confirmed through the rice root protoplast system. OsRopGEF3 was mainly located in the PM, whereas OsRac3 was located in the PM and the cytoplasm (Figures 7A–C). Next, the interaction of the two proteins was verified through BiFC in rice root cells. The Venus signal was observed in the PM, as shown by the BiFC results in tobacco (Figures 7D–F). Although we did not confirm the exact position of the two proteins in the rice root hair cells, our data suggest that the two proteins were colocalized in the root cells and interacted in the PM.

### OsRac3 Interacts With the N-Terminus of OsRBOH5

Significant ROS decrease in the *osropgef3* root hairs may be due to the functional failure of OsRac3. ROP/Rac binds to multiple proteins and is involved in various mechanisms. Among these roles, ROP/Rac has been reported to bind to RBOH, which produces ROS inside plant cells. Three RBOH genes are highly expressed in the root hairs of rice: *OsRBOH2*, *OsRBOH3*, and *OsRBOH5* (Kim et al., 2019). Accordingly, we aimed to detect OsRBOH, an interaction partner of OsRac3, using Y2H. The full CDS of OsRac3 and N-terminal region of OsRBOH were combined with the prey and bait domain. As a result, OsRac3 weakly interacted with the N-terminus of OsRBOH5 in the yeast system (Figure 5F). Furthermore, to confirm their interaction inside the rice cells, BiFC was performed in root protoplast cells. When both constructs were coexpressed within the protoplast, a strong clear signal was observed in the PM (Figures 7G–I). These results indicate that OsRac3 interacts with the N-terminus of OsRBOH5 in the PM of rice root cells.

## Discussion

In this study, we confirmed that *OsRopGEF3*, among the OsRopGEF family members, plays a key role in root hair development. When homozygous loss-of-function mutants were generated for *OsRopGEF3* using the CRISPR-Cas9 system, *osropgef3-1* and *osropgef3-2* showed abnormal phenotypes with root hair length decrease, root hair width increase, and wavy phenotype. Of the phenotypes in *osropgef3*, increased root hair width and wavy phenotype are assumed to be related to *OsRac3*. Previous studies have shown that changes in the activity of ROP/Rac induce abnormal phenotypes in root hairs. Supercentipede 1 (SCN1) is a RhoGTPase GDP dissociation inhibitor (RhoGDI) that negatively regulates ROP/Rac activity. The homozygous loss-of-function mutant of *SCN1* has a branched root hair phenotype (Parker et al., 2000), suggesting that the phenotypic change is due to abnormal ROP activity. Furthermore, mutations in both tip growth defective 1 (TIP1) and pluripetala (PLP) genes (*plp-3 tip1-4*) resulted in decreased root hair length and multiple root hair axes. These phenotypes are due to cytoplasmic accumulation and instability of ROP2 in the root hair apex. Interestingly, the single mutant of the PLP gene shows significantly reduced cytoplasmic ROS and abnormal actin dynamics (Chai et al., 2016). Swollen root hairs were mainly observed for the mutant of the TIP1 gene, a palmitoyl transferase (Hemsley et al., 2005). The loss-of-function mutants for both TIP1 and root hair defective 1 (RHD1) genes had huge swollen root hairs (Parker et al., 2000). It is predicted that the mutant has defective protein palmitoylation and cell wall synthesis and is related to ROP/Rac because palmitoylation of AtROP7, AtROP8, and AtROP10 is required for their proper functioning in the cells (Lavy et al., 2002).

Although ROP/Rac has an important function in root hair elongation, RopGEF is also essential because ROP/Rac cannot function normally without RopGEF. Since the GFP signal was observed in the gain-of-function line (*pOsRopGEF3:OsRopGEF3-GFP*), there is no doubt about the overexpression of this gene. However, this transgenic line showed no significant differences in the length and width of the root hair compared with those of the wild-type plant. This result suggests that OsRopGEF3 and OsRac3 proteins cannot work alone in root hair development. In *osropgef3*, OsRac3 cannot be activated to its GTP-binding form because OsRopGEF3 is absent, thereby affecting various downstream events involved in root hair development in the mutant. However, even in the presence of excessive OsRopGEF3, no phenotypic changes occurred in *osropgef3* due to the limited amount of OsRac3.

Another consideration is the subcellular localization of OsRopGEF3. According to protein domain analysis, OsRopGEF3 does not have a transmembrane domain. Nevertheless, the reason for its location in the PM is expected to be due to its interaction with other transmembrane proteins. RopGEF proteins have preserved serine residues in the C-terminus and have self-inhibition mechanisms involving interaction with the plant-specific ROP nucleotide exchanger (PRONE) domain of receptor-like kinase (RLK) (Fodor-Dunai et al., 2011; Craddock et al., 2012). Since a PRONE domain exists in OsRopGEF3, it is predicted that OsRopGEF3 might interact with root hair RLK. In the BiFC analysis of OsRopGEF3 with OsRac3 in tobacco leaf cells and rice root protoplasts, the YFP signal was located in the PM. These results indicate that OsRopGEF3 can also attach to RLK during its interaction with OsRac3 or does not bind directly but forms complex with other proteins (Akamatsu et al., 2015). Although OsRac3 is localized at the membrane near the cytoplasm when it is expressed alone, the BiFC signal is a meaningful demonstration of the interaction of OsRopGEF3 and OsRac3. However, our results do not provide direct evidence of colocalization or interaction between OsRopGEF3 and OsRac3 inside rice root hairs, thereby warranting further investigation.

Overall, this research aimed to emphasize the relationship of OsRopGEF3–OsRac3–OsRBOH5. The *osropgef3* mutant has significantly reduced ROS in its root hairs, and OsRac3 interacts with OsRBOH5. Based on previous studies, it is known that the activation of ROS in root hairs is mainly regulated by root hair defective-six like (RSL) and root hair-defective (RHD), which are induced by auxin treatment. It is well known that the RSL transcription factor upregulates RBOH, which produces ROS, and is involved in root hair growth (Mangano et al., 2017). However, the OsRBOH5 gene did not show any expression change in root hair tissues in response to the exogenous auxin treatment (Kim et al., 2019). Due to the lack of functional analysis of OsRac3 and OsRBOH5, our results cannot clearly demonstrate the relationship between ROS and root hair development in rice. However, this suggests that OsRBOH5 may not be affected by the transcription factor RSL and may function differently from the Auxin-IAA-RSL-RBOH mechanism. A functional study of OsRBOH5 will be needed to see if ROS produced by OsRBOH5 can affect root hair growth.

## Conclusion

**FIGURE 8:**
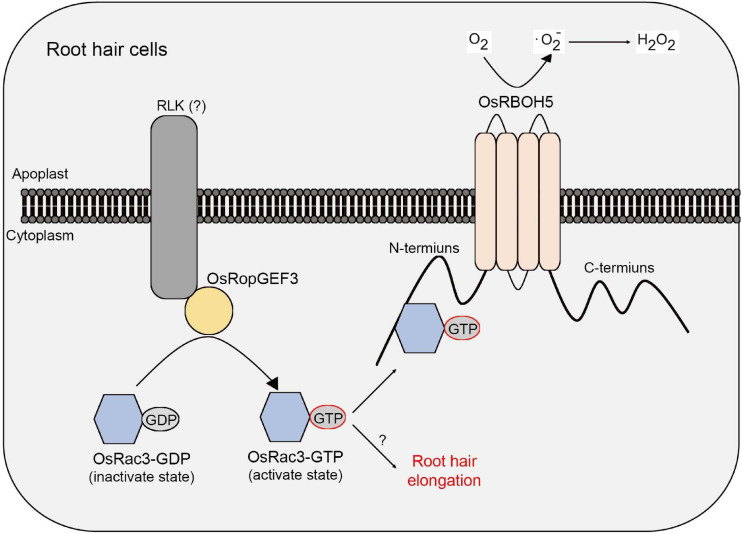
Models of OsRopGEF, OsRac, and OsRBOH proteins inside the rice root hair cells. Based on the PM, the upper part is the apoplast and the lower part is the cytoplasm. OsRopGEF3 localizes to the PM. However, since OsRopGEF3 does not have a transmembrane domain, it is expected to interact with RLK using the PRONE domain. OsRopGEF3 interacts with OsRac3 near the PM to change GDP to GTP. OsRac3-GTP was bound to the N-terminal of OsRBOH5 and resulted in ROS generation in the apoplast. Furthermore, OsRac3 can regulate root hair elongation. However, the specific pathway remains unknown.

This study demonstrates a correlation between RopGEF and ROP/Rac protein in rice root hair cells. Through a meta-expression analysis based on public microarray data, RT-qPCR, and GUS reporter system, it was demonstrated that *OsRopGEF3* is highly expressed in rice root hairs. Gain-of-function mutants tagged with fluorescent protein revealed that OsRopGEF3 was mainly located in the apical dome of the growing root hairs. In addition, the loss-of-function mutants, which were generated using the CRISPR/Cas9 system, showed a short and thick root hair phenotype. Furthermore, through Y2H and BiFC experiments, OsRac3 protein was identified as an interacting partner of OsRopGEF3. OsRac3 protein interacts with the N-terminus domain of OsRBOH5. Our results indicate that OsRopGEF3 interacts with OsRac3 protein and then exchanges GDP with GTP to activate OsRac3 in rice root hair cells. Furthermore, the activated form of OsRac3 protein interacts with OsRBOH5 to produce ROS (Figure 8).

## Data Availability Statement

The datasets presented in this study can be found in online repositories. The names of the repository/repositories and accession number(s) can be found in the article/Supplementary Material.

## Author Contributions

E-JK, Y-JK, and K-HJ conceived and designed the experiments, analyzed the data, and wrote the manuscript. E-JK, W-JH, and WT contributed to the experiments. GA and S-TK contributed to manuscript revision. All authors contributed to the article and approved the submitted version.

## Conflict of Interest

The authors declare that the research was conducted in the absence of any commercial or financial relationships that could be construed as a potential conflict of interest.

## Footnotes

1 http://rice.plantbiology.msu.edu/

2 http://www.cbs.dtu.dk/services/TMHMM/

3 https://pfam.xfam.org/

4 https://dna.macrogen.com

5 https://www.ncbi.nlm.nih.gov/geo/

## Supplementary Material

Supplementary Figure 1 Transmembrane domain analysis of *OsRopGEF3*, *OsRac3*, and *OsRBOH5*.

Supplementary Figure 2 Fluorescence intensity comparison of ROS staining.

Supplementary Figure 3 Identification of OsRac gene expression using qRT-PCR.

Supplementary Figure 4 Induction of plasmolysis in tobacco leaf epidermal cells by treatment with 1M NaCl. OsRopGEF3-eGFP and OsRac3-eGFP were individually transiently expressed in cells.

Supplementary Figure 5 Transient expression pattern of p35S-eGFP (empty vector) in tobacco leaf cells.

Supplementary Table 1 Primer sequences used in this study.
