# Supplementary figures and images for: Interaction of OsRopGEF3 Protein With OsRac3 to Regulate Root Hair Elongation and Reactive Oxygen Species Formation in Rice (Oryza sativa)
Source: Front Plant Sci. 2021 May 25;12:661352. doi: 10.3389/fpls.2021.661352 (PMC8185220; doi:10.3389/fpls.2021.661352)

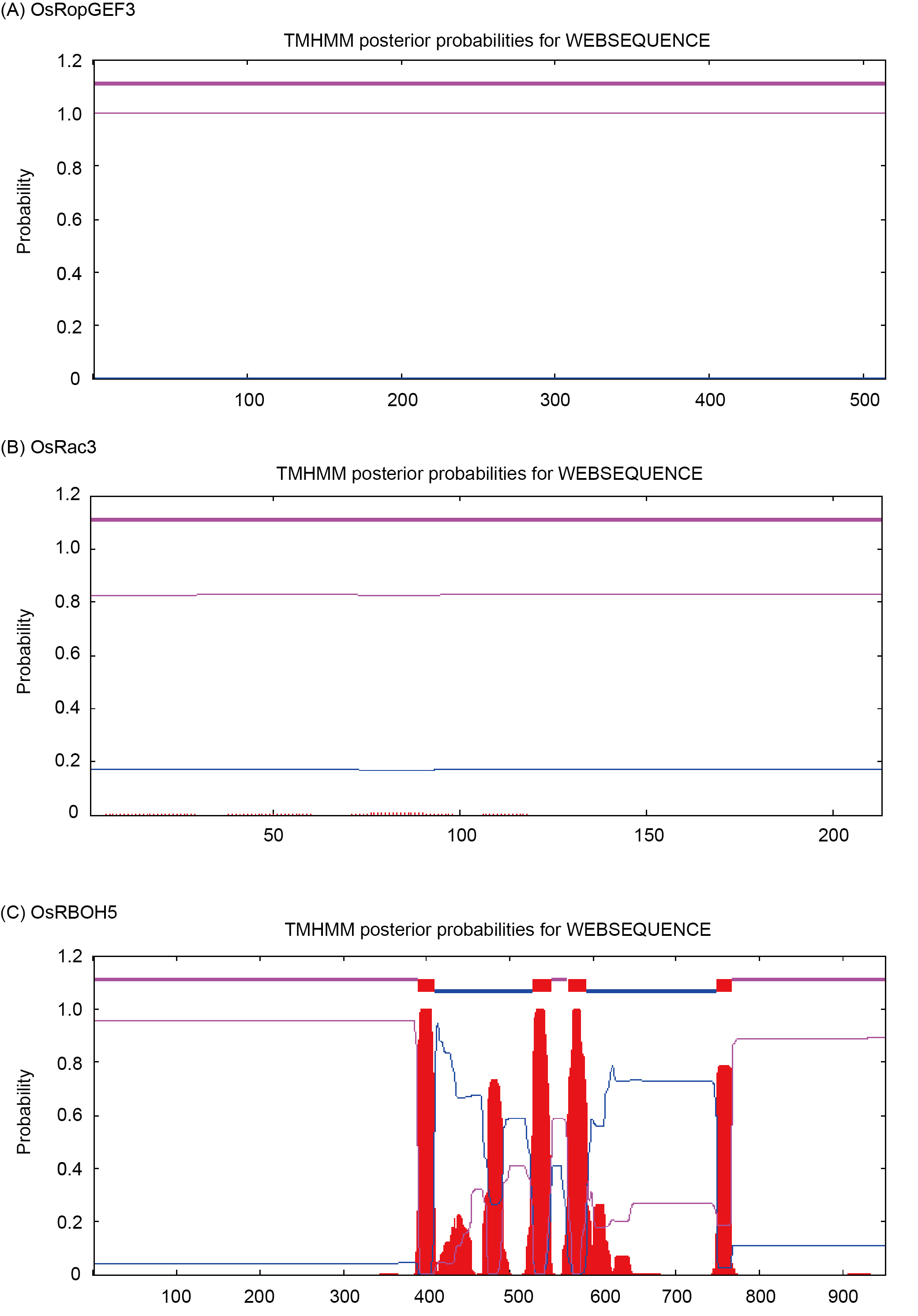

Supplement: Supplementary Figure 1 — Transmembrane domain analysis of OsRopGEF3, OsRac3, and OsRBOH5. [file Image_1.JPEG]

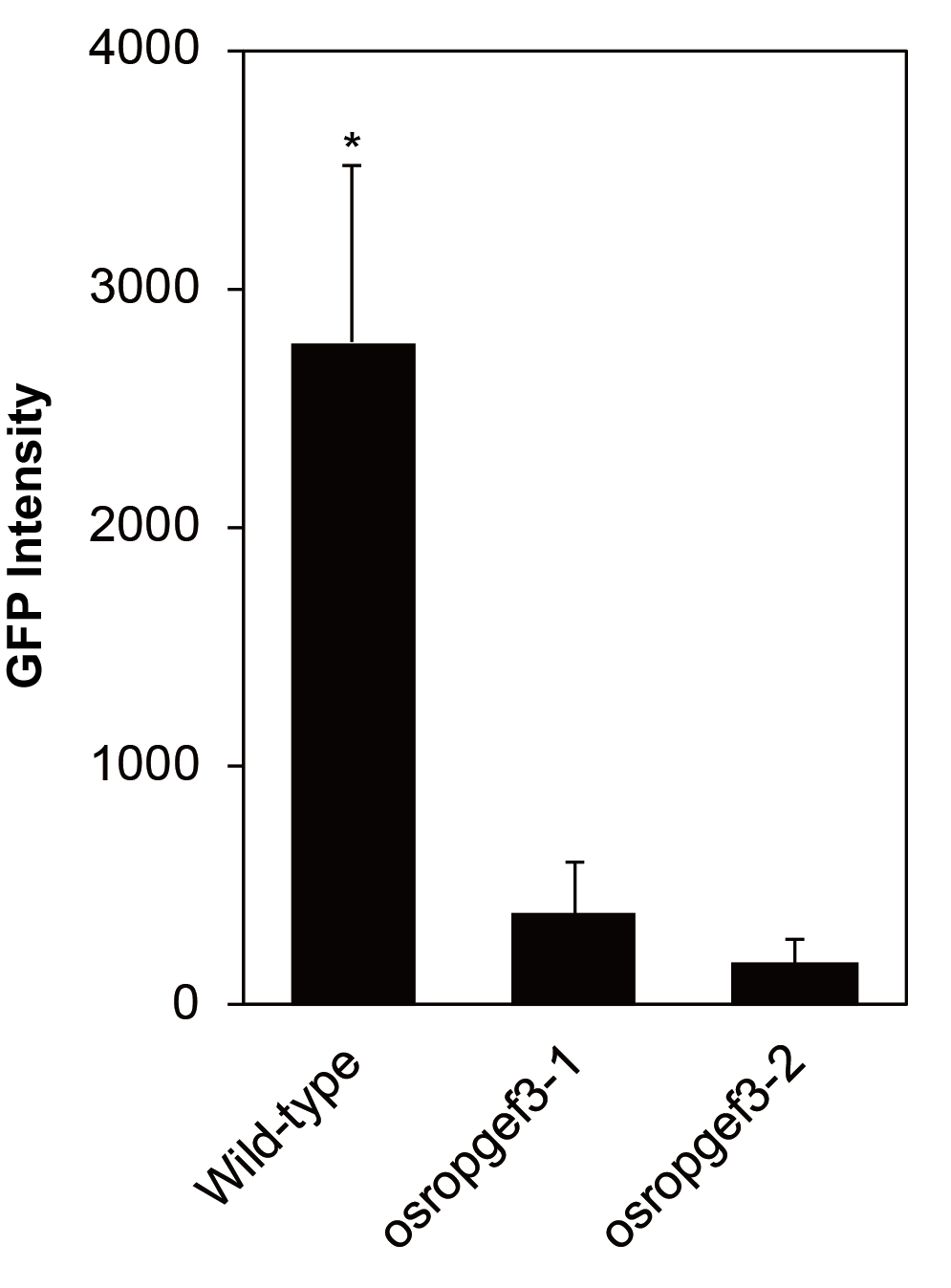

Supplement: Supplementary Figure 2 — Fluorescence intensity comparison of ROS staining. [file Image_2.JPEG]

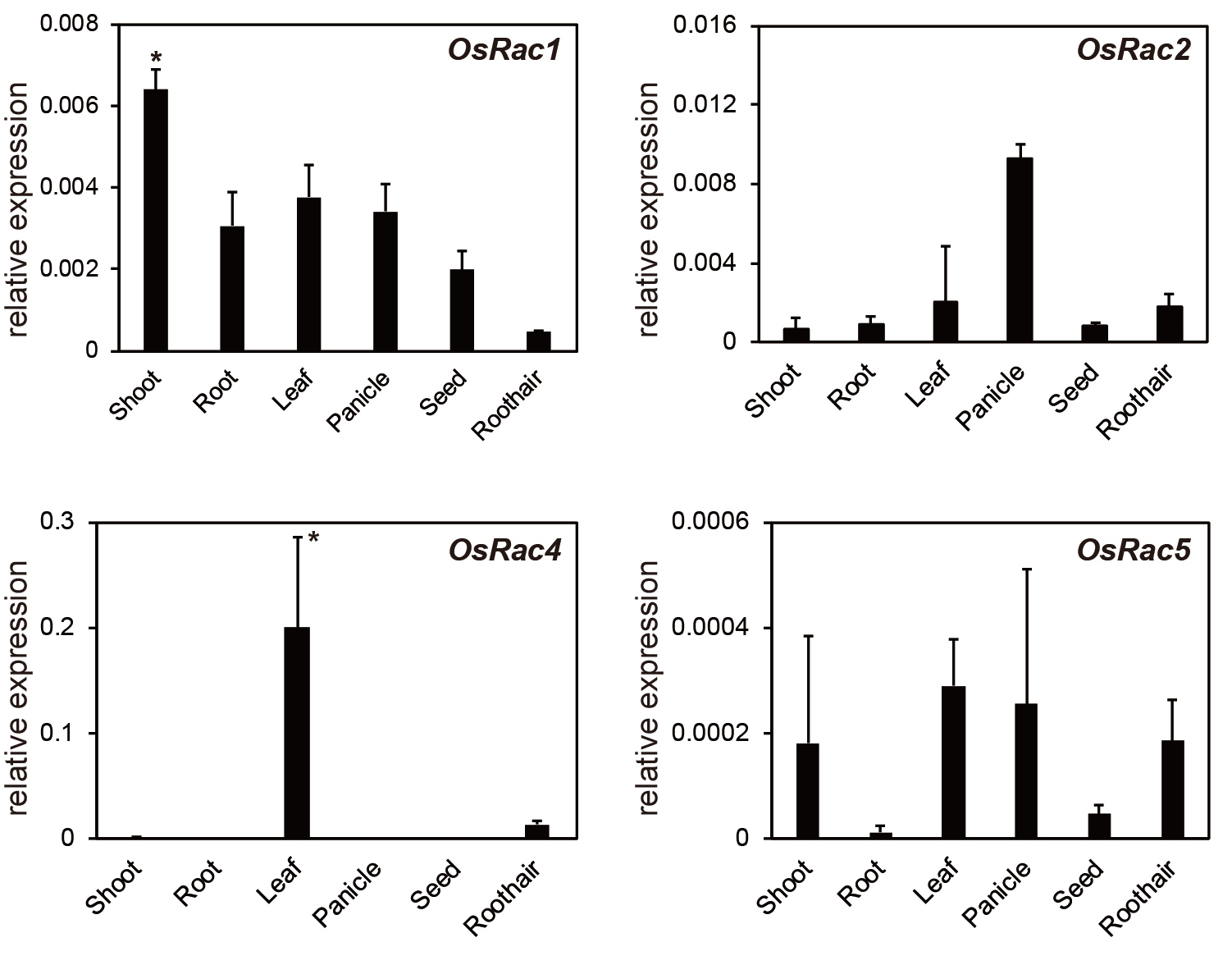

Supplement: Supplementary Figure 3 — Identification of OsRac gene expression using qRT-PCR. [file Image_3.JPEG]

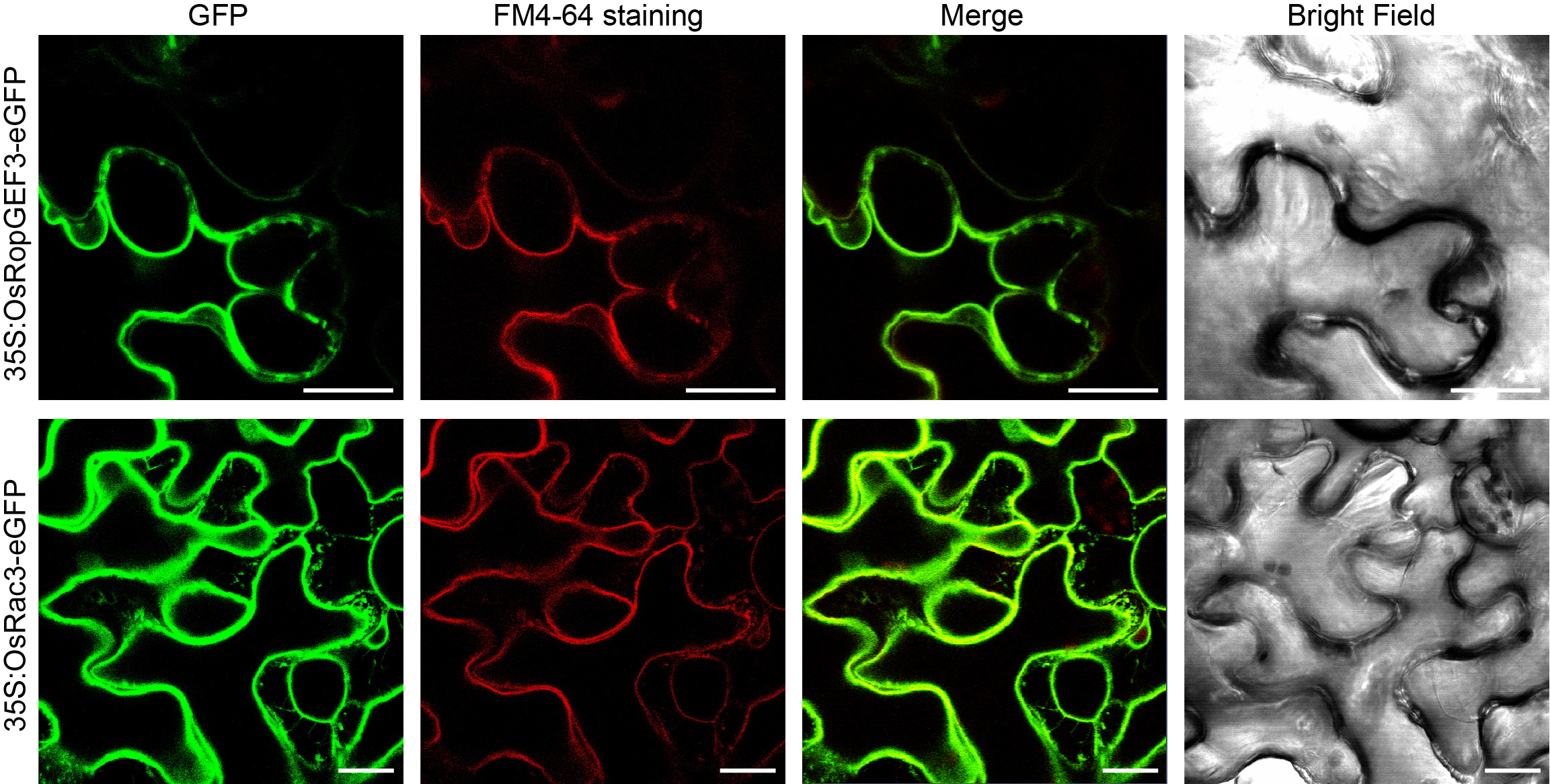

Supplement: Supplementary Figure 4 — Induction of plasmolysis in tobacco leaf epidermal cells by treatment with 1M NaCl. OsRopGEF3-eGFP and OsRac3-eGFP were individually transiently expressed in cells. [file Image_4.JPEG]

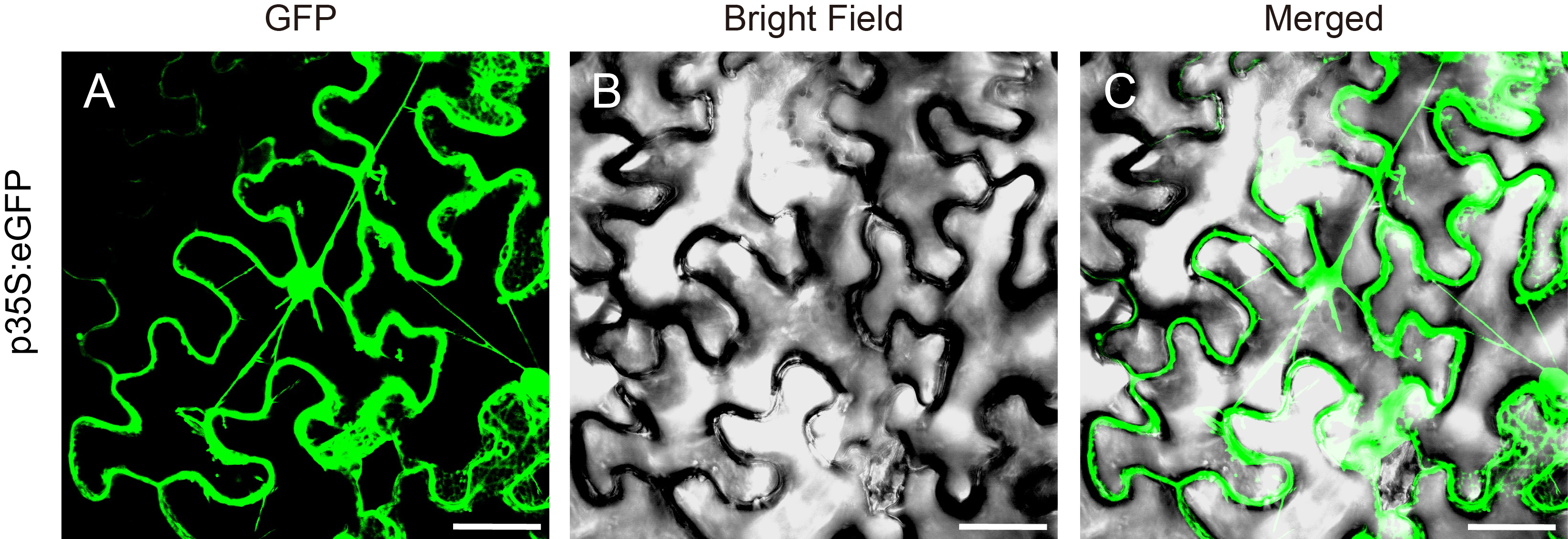

Supplement: Supplementary Figure 5 — Transient expression pattern of p35S-eGFP (empty vector) in tobacco leaf cells. [file Image_5.JPEG]
